# An Epistemological Perspective of Integrated Multidisciplinary Treatment When Dealing With Infertile Women With a Parenthood Goal: The Importance of Matterpsychic Perspective

**DOI:** 10.3389/fpsyg.2021.634028

**Published:** 2021-03-08

**Authors:** Francesca Natascia Vasta, Raffaella Girelli

**Affiliations:** ^1^Confederation of Italian Organizations for Analytical Research on Groups – Research Board, Rome, Italy; ^2^Confederation of Italian Organizations for Analytical Research on Groups, Rome, Italy

**Keywords:** female infertility-sterility, socio-anthropological level, condition of matterpsychic, assisted reproductive technology treatments, integrated multidisciplinary treatment

## Abstract

This article proposes a multidisciplinary work perspective on couples who undergo assisted reproductive technology (ART) treatments, with particular attention paid to the treatment of women. The epistemological references underlying a vision of infertility and sterility that respect the psyche–soma unity of the person are illustrated: the biopsychosocial model and the psychoneuroimmunology and psychosomatic contemporary models of health and illness. Based on clinical experience in a private and institutional setting, different steps in the course of treatment are described with the related areas of psychological work: from the search for pregnancy to the choice of adhering to an ART treatment, to conception, up to delivery and beyond. The implications of the work are targeted at a better qualification of psychological interventions in this specific multidisciplinary area.

“The old healer of the soul said:

It’s not your back that hurts, but the burden.
It’s not your eyes that hurt, but injustice.
It’s not your head that hurts, it’s your thoughts.
Not the throat, but what you don’t express or say with anger.
Not the stomach hurts, but what the soul does not digest.

It’s not the liver that hurt, but the anger.
It’s not your heart that hurts, but love.
And it is love itself,
that contains the most powerful medicine.”
The Old Healer to the Soul by Ada Luz Marquez

“Feelings are not an independent production of the brain, but the result of a cooperative alliance between the body and the brain […] We must point out that a collaboration between nervous systems and bodies was necessary to generate human minds, and that minds have manifested in organisms not already isolated, but which were part of a social context” (Damasio, 2018, p. 22, 88).

## Introduction

Our epistemological position contains and develops the principles of the biopsychosocial model, of the psychoneuroimmunological model, and of contemporary psychosomatics regarding the interpretation of female infertility–sterility, underlining that the emotional, social, and medical–biological aspects of the person cannot be separated (subsequently defined as a whole “matterpsychic”).

In particular, following this perspective, the interventions for the woman (and the couple) who undergoes assisted reproductive technology (ART) treatments need to be organized through an integrated multidisciplinary work. This is revealed more clearly considering that the presence of infertility manifests at a specific time in the cycle of life, not through pathological symptoms but with the failure to achieve a desire for the individual and the couple (Greil et al., 2011).

According to the biopsychosocial model developed by Engel (1977) on the basis of the multidimensional concept of health described in 1947 by the World Health Organization (WHO), the afflicted individual was placed at the center of a system influenced by various factors. To comprehend and treat, the doctor not only has to look at the problems of functions and organs but must also pay attention to the psychological, social, and familial aspects of the individual, which interact with each other and are able to influence the evolution of the disease. The medical aspects relating to infertility were the first to receive a wide attention (da Motta and Serafini, 2002; Lindsay and Vitrikas, 2015; Cunningham, 2017); the psychological ones were later considered in various terms, among which the effects of the diagnosis of infertility and the application of specific infertility counseling (Boivin et al., 2001; Boivin and Gameiro, 2015). We have recently begun to study and deepen social aspects such as the advanced age of women at the moment of conception, lifestyle habits such as smoking and obesity, the fact of being single or part of a homosexual couple, and the donation of gametes and management of the unveiling to their family network and subsequently to the children born from this process (Dhillon et al., 2000; Cousineau and Domar, 2007; Mohiyiddeen and Cerra, 2017; Walentynowicz-Moryl, 2020).

Psychoneuroimmunology informs us of the mutual interaction between behavior, mental activity, the nervous system, the endocrine system, and the immune response of human beings (Procaccini et al., 2014; Bottaccioli and Bottaccioli, 2017). Here, we want to recall some of the discoveries that have contributed to the foundation of this discipline such as Candace Pert’s studies on emotions (1997), in which she describes how neurotransmitters, called peptides, carry emotional messages. These messages change the chemistry of the cells in our body. Neuropeptides and their receptors are the biological substrate of emotions and are in constant communication with the immune system, the system, as it is commonly known, through which health and disease are created.

For this reason, we can state that contemporary psychosomatics has overcome the classical conception according to which something that happens in the mind can reflect on the body, which thus supported a clear distinction between psychosomatic illnesses and those of organic origin. This distinction excluded most of the somatic pathology from psychological intervention, and it proposed the possibility of obtaining therapeutic results in some diseases just through psychological intervention, with disappointing results. Such perspective also referred to an isolated individual and paid no attention to the relational context, while in contemporary research, this dimension for the health and illness of the person is central (Solano, 2016, 2018).

We suggest using a multidisciplinary approach and treatment, which not only aims at obtaining pregnancy but also guarantees the development of a disciplined clinical practice that includes a comparison and a systematic dialogue in correspondence of all the steps of the treatment between the various professional figures involved (gynecologist/andrologist; biologist; geneticist/genetic counselor; psychologist/psychotherapist; obstetrician; nurse; cultural mediator; neonatologist pediatrician; pediatric nurse).

We also specify that we always address our psychological treatment to the couple.^1^ In this work, we have decided to deal specifically with the condition of infertile women (Namdar et al., 2017; Li et al., 2019), on which we are currently focusing our studies (Vasta and Girelli, 2019; Vasta, 2020a).

Table 1 presents both the challenges and tasks that the woman/couple have to face at each stage and the competences and duties of the carers as a working multidisciplinary team.

**TABLE 1:** The stages of woman/couple’s journey in ART treatments.

Stages of the journey	Challenges and goals for women	Tasks of the woman/couple (to be pursued with the support of a multidisciplinary team)	Health professionals involved (the caregiver of the multidisciplinary working team)	Required skills of caregivers (in addition to general technical-professional ones)
Infertility/sterility diagnosis	Addressing the identity crisis following the diagnosis (see par. 5.1).	• Acceptance and elaboration of the diagnosis. • Gather clear information on possible treatments. • Get rid of any prejudices about the treatment that has to be undertaken or however avoid them if they dominate the decision-making field. • Make decisions on whether or not to start the path with awareness and sense of responsibility shared with the caregivers.	• Gynecologist/Andrologist • Psychologist/Psychotherapist • Possible cultural mediator • Other doctor of reference of the patient for any previous pathology.	• Ability to communicate with colleagues of different professions and to work as a multidisciplinary team. • Specific training on this clinical area. • Assume and maintain a global vision of the patient as a mind-body unit inserted in his socio-anthropological context (see par. 2 e 4). • Empathic communication with the patient (in the various steps from the diagnosis onwards). • Take responsibility for accompanying the patient as a working multidisciplinary team throughout the treatment (until after childbirth).
ART treatment start-up	• Relying on the multidisciplinary team. • Taking care of yourself globally, as a mind-body unit (a condition of matterpsychic: see par. 2). • Keep the focus on the present moment, without going too far in the future or the past.	• Sign up to various medical appointments. • Establish a permanent psychological support for the couple with the psychologist/psychotherapist of the multidisciplinary team. • Attend your own socio-emotional network (avoid isolating along the way). • Use the psychological support space to process any early failures that occurred during treatment and comprehend if and when to interrupt the treatments.	• Biologist • Gynecologist/Andrologist • Geneticist/Genetic Counselor • Psychologist/Psychotherapist	• Maintain all the above. • Schedule periodic meetings as a multidisciplinary team for the entire duration of the treatment in which discuss and make shared decisions.
Pregnancy	• Accept that the journey in ART treatment is not over yet, even if conceiving has finally taken place. • Continue to take care of yourself in a global way, with additional self-protective measures. • Manage the fears and anxieties related to the new psychophysical condition.	• Continue the psychological work to address the fears associated with the various stages of pregnancy, childbirth and following the birth.		
Delivery and birth	• Consciously choose the modality of giving birth (natural childbirth or caesarean section: see par. “Achievement of pregnancy and child birth: psychophysical transformations”) • After birth, establish a relationship with the real child (who is not the one imagined). • Maintain confidence in your parenting skills in the first months of life, especially in front of the first evolutionary challenges (breastfeeding, sleep-wake rhythms, prime first baby food, etc.).		• In addition to the aforementioned careers: • Obstetrician • Nurse • Pediatric Nurse • Neonatologist pediatrician.	• It is useful for the psychologist/psychotherapist to organize a pre-delivery meeting with the obstetrician and the nurse to describe the psychological condition of the woman and the couple, informing these professionals on the importance of empathic communication. • The same can be done immediately after the birth with the neonatologist pediatrician and the pediatric nurse.

A clinical case is illustrated (see section “Discussion”) in order to show our therapeutic intervention and perspective and to offer useful evidence for further implications.

## Female Infertility and Sterility^2^: A Condition of Matterpsychic

In an effort to support this unity also on an epistemological level, we use in our work the term matterpsychic of philosophical origin (Pauli, 1952; Sparzani and Panepucci, 2016). We therefore assume a conception where body and mind do not exist in a specific and distinct form in relation to the whole organism, rather they are two categories that have to do with the vertex from which the observer sets himself. This conception has ancient roots, as the philosopher Michel Foucault (1963) claims when retracing the history of medicine. It was already present in the thought of the philosopher Baruch Spinoza.^3^ We must therefore not build from scratch but recover a concept of disease and cure.

In this perspective, certain steps of clinical relevance are explored: the diagnosis of infertility/sterility, the failure of ART treatments, pregnancy achievement, gestation, and childbirth. In this way, we underline that the discovery of infertility–sterility does not represent a single event; rather, it is configured as the beginning of a series of evolutionary challenges which involve the individual and the couple in their body/mind uniqueness.

## Medicalization of Sterility: The Dis-Integration of Matterpsychic

Medicalizing means considering the patient’s body as an object of care, simplifying the complex nature of the sick person and creating emotional distance between the caregiver and the person being treated (Colucci, 2006; Vernero, 2017). The patient’s needs are reduced to malfunctions of the organism (Ongaro Basaglia, 2012), and medicine in this perspective is seen as a science endowed with technological and pharmacological power rather than *therapeutic* power in the etymological sense of the term. The word therapist comes from the Greek *θεραπεýω* (*Terapeuo*) and means “I am at the service of.” This position of medicalizing the illness is opposite to the one that, already in the last century, the psychoanalyst Balint (1957) supported when speaking of the doctor as the (principal) drug for the patient.

We must therefore ask ourselves whether it is possible to treat the problem of infertility/sterility and the related course of treatment only from a medical point of view, emphasizing the medicalization^4^ of infertility/sterility or providing psychological treatment only as a path on a parallel track that proceeds on its own. As anticipated, this article aims to offer a contribution to the formulation of an articulated answer to the question. With this objective, we intend to draw the attention to the socio-anthropological aspects of the problem (Khetarpal and Singh, 2012; Hocaoglu, 2018; Vasta, 2020a).

## Socio-Anthropological Level: Opacity vs. Permeability of Care Contexts

Each person is immersed in his own historical–anthropological and social contexts of reference. This context in our perspective does not represent a single frame in the life of the person but shapes an identity component and influences his choices (Pacilli, 2019; Vasta, 2020b).

Without no claim to be exhaustive, we report some aspects of this context that have appeared relevant to us while following couples who receive ART treatments.

For our argument, it is useful to remember that in Italy the law prevented gamete donation until 2015. The prohibition has contributed to the consolidation of social prejudices towards this choice. Since 2017, medically assisted procreation has been included in the essential levels of assistance (LEA).^5^

Another component of the Italian socio-cultural context may also make complex both the choice of relying on medically assisted fertilization, in particular gamete donation, and the management of the subsequent steps. Italy has a culture, in the broad sense of the term, which is influenced by the Catholic matrix, also for historical reasons. In our clinical experience, both in the institutional area and in private practice, this element has been required to be taken into consideration as much as other aspects of more obvious attention (from the doctor, for example, the age of the couple, rather than familiarity with certain organic pathologies), in line with the indications of the ESHRE guidelines for routine psychosocial care in infertility and medically assisted reproduction (Gameiro et al., 2015).

In the end, the COVID-19 pandemic has brought with it a whole series of constraints in the protocols, for example, the start-up times of a new ART treatment attempt forcedly anticipated by the fear of a closure of the health center for a possible second *lockdown* and the impossibility of having close relatives during pregnancy or the child’s father in the delivery room. All this has generated negative feelings, particularly with regard to the closure of the centers during the *lockdown*; the literature reports feelings of fear, uncertainty, frustration, and anger and a sense of injustice suffered (Boivin et al., 2020).

Therefore, even considering the social level of the problem, an indispensable factor emerges for the effectiveness of the entire treatment process: the different professionals thinking together about the times and methods of the steps to be taken (Di Trani, 2018).

## The Female’s Journey in Art Treatments: Suspended Identities

We have to consider the different evolutionary challenges faced by the couple during treatment, paying attention to the position of the woman, in line with the purpose of the article.

First of all, we recall that the aforementioned ESHRE guidelines (Guidelines for Counseling in Infertility, 2001) involve different types of “patient-centered care” psychosocial intervention for ART treatments: informative counseling on the implications of treatment; psychological support counseling for specific critical steps—for example, waiting for the results of the implant or for the preliminary examination ones (Boivin and Lancastle, 2010; Cipolletta and Faccio, 2013; Ockhuijsen et al., 2013), a phase of intense care, the failure of a cycle of treatment, and the choice of whether or not to continue along the medical course—which aims to mobilize the couple’s resources and define strategies to cope with stress; and psychotherapeutic intervention, in the presence of diagnosed disorders of the individual or couple.

### Diagnosis vs. Acceptance/Elaboration/Decision

First of all, we underline that motherhood represents a complex human condition, not attributable only to the physical, psychic, and cultural components (Schirone, 2013). Stern (1995) and Stern et al. (1998) has highlighted the relationship between the identity shift and becoming a mother. Subsequent research has deepened the qualitative characteristics of identity changes of women in the process of becoming mothers (Laney et al., 2014, 2015); the role of cultural factors in facilitating or not the passage of identity (Märtsin, 2018; Gardner et al., 2020); and how the possibility of reorganizing the attachment during the transition to motherhood by mothers with unresolved traumatic past experiences is a key factor for the quality of care provided to the child and also for the type of attachment that he will develop (Iyengar et al., 2019).

The search for a pregnancy that results in the diagnosis of infertility and even more so of sterility can cause a crisis of such magnitude that it can be defined as an “identity” crisis (Thorn, 2009). It is the body image that has been damaged, with repercussions exactly on the level of identity and gender identity (Salerno and Piccolo, 2006; Rosner, 2012; Patel et al., 2018). The experience of identity loss takes place on several levels: in genetic continuity; in the image of oneself as a fertile person; and regarding the possibility of pregnancy and childbirth. The effects on each individual are of course different. However, literature has highlighted how the diagnosis is accompanied by emotional experiences such as shock, rejection, frustration, and feelings of inadequacy.

It is not only the woman, but also the couple, that goes through a suffering triggered by the failure of carrying out a project related to motherhood/fatherhood, which involves psychological well-being, marital relationship, sexual relationship, and quality of life. A review of 20 articles in English (2000–2014) by Luk and Loke (2015) and subsequent studies (Luk and Loke, 2019) highlight that infertility has a negative effect on the psychological well-being and sexual relationships of couples. A recent study on Chinese couples with infertility in Hong Kong has demonstrated the negative association between quality of life of infertile couples and infertility-related stress and the role of family sense of coherence in promoting infertile couples’ well-being (Ngai and Loke, 2021).

Furthermore, it has been proved that the way a partner reacts to infertility can have a great influence on the other partner (Berghuis and Stanton, 2002; Chaves et al., 2019; Ha and Ban, 2020); also, a positive correlation was found between the anxious and/or avoidant attachment styles of the two partners and the perceived level of distressing infertility on the other (Van den Broeck et al., 2010; Donarelli et al., 2012, 2016). Even when studies do not allow us to find a causal relationship, they still underline the importance of considering attachment patterns and related abilities of emotion regulation in psychological counseling for promoting couples’ health (Moura-Ramos et al., 2017).

The psychological and psychosocial interventions, based on the multidisciplinary dialogue with the doctor who performed the diagnosis, in this phase are aimed at preventing the development of psychological distress (Van den Broeck et al., 2010; Frederiksen et al., 2015; Molgora et al., 2020) and analyzing with the couple the scenario in which the choice to arrive at a center for ART treatments occurs. Inside (or outside) the couple, by whom is the decision made? How long after the diagnosis? Was the couple alone at this juncture, or were they able to deal with family and/or friends? Clinical experience confirms that depending on the answer to these questions, the subsequent challenges in the course of treatment within the center will be experienced by the couple as more or less difficult. We would like to emphasize that the diagnosis of infertility has an impact on the dyad, on the couple, regardless of whomever of the two partners received it. In any case, the positive influence of partner and family support is underlined in literature (Martins et al., 2014a, b), as well as the marital stability for lower stress levels of the couple both initial and subsequent (Martins et al., 2014a, b). Through research on psychological counseling for infertility, it has been demonstrated that the couple responds to treatment as a whole; in fact, the effects of psychological work result as a change in the couple’s relational dynamics, not just as a change in the individual partner (Donarelli et al., 2019). As a confirmation of this, other studies have shown that the capacity of the partners to face this experience is the result of both individual and relational coping strategies (Zurlo et al., 2018, 2019, 2020; Molgora et al., 2019).

Furthermore, the health protocols of ART treatments include the precise scanning of execution times and techniques. Considering only the physical level, through the development of biotechnology, fertilization can be perceived as a definitive solution to suffering, and the fertility center or clinic where they arrive can be invested with miraculous expectations. If it is the woman who is identified as the one who cannot achieve conception, the initiation of the protocol in order to have a biological child becomes the main objective. We immediately start to face statistics about the chances of success related to age, calendars and drug administration, endometrial preparation cycles for implantation, self-monitoring of the body experience to detect signs of pregnancy, expectations, analysis, and results.

In this way, the protocols force one to concentrate on the soma (excluding the level of matterpsychic), running the risk of leaving the thought in the background. It is precisely that dangerous mechanism that we have defined “dis-integration of psychic matter”: the biological aspects are isolated from the affective and body aspects. Instead, we know from the studies carried out that the treatment cycles are characterized by hope, expectation, and stress, followed by disillusionment, sadness, and other negative emotions (Mohiyiddeen and Cerra, 2017).

Once again, we reaffirm that it can be the multidisciplinary team, cohesive and with a good internal dialogue, that makes sure that the mind–body splitting mechanism does not take over.

### Achievement of Pregnancy and Child Birth: Psychophysical Transformations

We now refer to the clinical experience with couples who, following the diagnosis of sterility, achieved a pregnancy after two or more cycles of gamete donation in ART treatment (in some cases after the failure with the homologue, which has represented a further critical step).

Achieving pregnancy involves different emotions: joy, surprise, hope, and fear (Boz et al., 2018).

The woman’s body becomes the container of the child’s body, and the pregnancy proceeds through the establishment of a psychobiological relationship.

From the beginning of conception, the woman undergoes medical procedures and the intake of medications to protect the pregnancy.

Even in this passage, if the problem dealt with by the couple up to now is seen only from the somatic point of view (medicalization of sterility), one might think that the couple has reached the resolution of the problem. Instead, we propose to consider conception and pregnancy from the perspective of the totality of the matterpsychic woman. This implies that the team has to undertake the preparation of appropriate settings to elaborate and support the changes and challenges taking place. If it is true that an important goal has been achieved, it is equally true (it is real) that complications can occur. When pregnancy presents complications (high blood pressure, shortening of the uterus neck, placental abruption, etc.), the woman can experience a strong sense of danger, sometimes guilt. In some cases, absolute rest may be prescribed. The constant worry regarding the evolution of pregnancy causes great stress, one can experience the feeling that the body betrays again, and we confront ourselves with new limits and attend the birth as a moment of relief and joy. Furthermore, even if the complication does not occur, we cannot think that all the past emotional history preceding conception can magically disappear with the arrival of pregnancy. Instead, we believe that it is a protective factor of pregnancy and of the subsequent development of the bond with your child to work on the awareness of the present moment and the path undertaken. Among the possible complications, an early (abrupt) interruption of pregnancy and premature birth can occur. We have described (Vasta et al., 2013; Vasta and Girelli, 2016) the psychological conditions of parents who suffer this potentially traumatic event, illustrating how the psychosocial intervention addressed to them, with the involvement of the healthcare staff of the neonatal intensive care unit (NICU) in which premature babies are hospitalized, can be a useful prevention and treatment tool for the new family unit. Here, we just remember that in literature, it has emerged that the risk of preterm birth in singleton pregnancies resulting from ART treatments is significantly greater than that in spontaneously conceived singletons (Cavoretto et al., 2018). However, also drawing on our 10-year clinical experience in the NICU, we believe that premature birth should be taken into consideration as a possible reality scenario after a course of ART treatment, especially if gamete donation was performed (Vasta and Girelli, 2017). Beyond the specific situation of prematurity, childbirth can represent a new trial that has to be dealt with. Through our clinical experience, we would want to bring attention to the wish of many women to give birth naturally, and not through a cesarean section. Natural childbirth is thus perceived as a proactive stance to facilitate their child’s entry into the world and seems to have the specific function of letting women regain the feeling of having a body tuned to their desires. However, this intention may not coincide with the most protective choice for the success of childbirth. For example, a rise in blood pressure may occur during pregnancy, and a cesarean section may be more suitable. If the woman/couple did not have the opportunity to consider these aspects with the psychologist during pregnancy, even if they were presented to her as statistically possible events by the doctor, she could experience the birth with a double trauma: that of the unexpected event (the increase in pressure) and that of the renunciation of natural childbirth.

### Breastfeeding: Acceptance and Relationship

Breastfeeding may also be specifically connoted as a challenge, as a space for the woman to recover the safety of her maternal function. Breastfeeding can, like natural childbirth, be loaded with expectations and functions. It is important to work on these aspects on a psychological level and to help the mother and the couple to focus on which elements the attachment relationship with the child is really based on. For example, it could happen that the mother cannot breastfeed regardless of her will and develops again a sense of guilt and inadequacy or that she adopts methods and times of breastfeeding that do not respond to an adequate tuning of the real needs of the child but rather to her own self-healing needs that we have already talked about. A recent study (Barrera et al., 2019) has emphasized how these mothers who conceive using ART treatments may breastfeed for shorter periods than mothers who conceive spontaneously, partially mediated by a likelihood of giving birth preterm or multiple birth. More research is needed to clarify these associations and to understand the intentions and barriers to breastfeeding among women who achieved pregnancies through ART treatments.

## Clinical Case^6^

Pia and Giorgio contacted me after getting my contact details from their gynecologist, Dr. R.

Dr. R. and I are part of an association of professionals (psychologists, psychotherapists, gynecologists, biologists, lawyers, social psychologists, and geneticists) who deal with ART treatments in Italy.

I inform the couple that my working method involves a constant dialogue and discussion with the gynecologist and therefore I ask to be able to contact Dr. R. before our meeting to have medical information on their situation. After receiving consent, I call her and I am told that the partners have undergone several cycles of homologous fertilization.

Moreover, Pia has just received the diagnosis of premature ovarian failure; the gynecologist thus proposed the possibility of pregnancy through egg donation and at the same time pointed out the usefulness of dealing with this step also on a psychological level. The couple seems surprised by the doctor’s proposal regarding psychological counseling.

Before starting the treatment with Dr. R., the couple had visited other two counseling centers, meeting doctors who had addressed the specific problem by showing general success/failure statistics and superficially informing them of the presence of a psychologist in the center.

Therefore, I meet with the couple, and we set up a five-session counseling meeting to analyze their questions.

The couple, 38 years old (Pia) and 39 years old (Giorgio), had tried for at least 2 years to have children naturally; she turned to ART treatments about 3 years ago. They are a solid couple who love each other a lot, and both have satisfying jobs and a good social network. They wish to have a child but are also aware and accept that this option may not occur.

In the course of counseling meetings, we explore together all the issues which surround gamete donation: the difficulty in accepting the diagnosis; the loss of security with respect to the parenting project; the discussion with the couple on how this decision can influence the bond between the partners; the (religious) prejudices of a part of her family of origin; thoughts about the future; and the possible sensations during pregnancy and after birth in the relationship with the child.

At the end of the counseling process, the couple chooses to follow the fertility treatment with egg donation. Pia and Giorgio appear united in facing the next challenges. The good basic bond between the partners has facilitated the couple’s psychological work aimed to take a common decision.

We part, I confirm my availability for the future, aware that the ART treatment path could also subsequently require the need for a listening space.

After a few months, in fact, I receive a call from Pia who tells me she is close to the transfer, and we decide to arrange some meetings. I contact the gynecologist for a discussion regarding the situation again. The gynecologist tells me that at this stage, she has received many phone calls from Pia. The woman has asked to speak to the biologist, calling him daily to find out how the embryo was developing. In agreement with Dr. R., I also contact the biologist for an exchange of views, and we decide that on the day of the transfer, we will be present together with the gynecologist to greet the couple.

After the transfer, Pia and Giorgio did not come to the scheduled meeting: Pia wants to rest until the day of the beta HCG levels. I suggested Pia contact me in order to handle the need of sharing her fear of the possible failure of the engraftment of the embryo. She resurfaces the memory of previous failed attempts. Pia is convinced that by staying at home, she can protect this engraftment.

After the analysis and the news of the pregnancy, we agree with the couple for a fortnightly meeting with the aim of continuing to support the couple in facing the subsequent steps and related anxieties.

For example, Pia is worried because now Dr. R. won’t follow the pregnancy but Dr. Z. of the University Hospital where Pia will give birth. We plan a periodic comparison between me and the gynecologist who follows the pregnancy.

Around the 8th week of pregnancy, Pia manifests a strong hyperemesis gravidarum. She is very depressed, and she is afraid to miscarry.

We resume meeting again at the 18th week, considering that Pia is feeling better. Unfortunately, around the seventh month, she experienced a threat of abortion and is hospitalized. I plan a couple of visits to Pia at the hospital. We only have two short sessions, but Pia on that occasion proves to be very grateful for seeing me there. She feels guilty for what is happening, and she fears that she has “crossed the line,” by not receiving the support of her family of origin which has never been fully convinced of her choices. Giorgio reminds her that they did it together, that he is present. Pia talks on the phone with her gynecologist, but now the doctors in the obstetric pathology department are taking care of her, not her gynecologist. She feels scared, in the hands of unknown doctors, she is very down in the dumps, and she is afraid of losing her pregnancy.

An emergency cesarean section is needed: the child, Pietro, will remain in sub-intensive care unit in the hospital for 4 weeks.

At that juncture, I introduced myself to the hospital staff, and I tried to present the history of Pia and Giorgio. This abrupt termination of pregnancy, premature birth, and the delicacy of the baby’s condition rendered the weeks of hospitalization particularly difficult. We have continued our meetings after discharge, facing the challenges as they arose: Pia’s mood swings, Giorgio’s tiredness (who in the moments that immediately followed the birth kept in touch with the doctors, encouraged Pia, but he himself felt very afraid for her and for the baby); breastfeeding; the reorganization of daily life; and Pietro’s first medical check-ups after his discharge. At this stage, I got in touch with the pediatric neonatologist to work together on supporting Pia’s parental function, severely tested by the precipitous events of the last part of her pregnancy and her son’s hospitalization. Today, Pietro is 2 years old, and he is a beautiful child; he has been walking since he was 18 months old, and at 20 months, he said his first word: mom.

### Discussion

We, as mental health professional specialists in this field, are aware that all new parents face challenges during the transition to parenthood (Doss and Rhoades, 2017). The case presented shows our attempt to work in an integrated manner in the private setting through the full involvement of the various professional figures responsible for the physical and mental care of women who intend to follow an ART treatment path.

In the light of this experience, at least two further aspects worth noting emerge, in addition to what has already been written in this work.

1. It is important that the psychologist adopt a flexible setting that always allows his presence next to the couple in critical moments. For example, when Pia does not want to leave home to go to the psychologist’s office, the therapist understands that there is no real need for Pia to stay at home in terms of physical health but rather that it is very important through that stage to satisfy Pia’s emotional need to protect the pregnancy in the only way that the woman has available (staying at home). In this sense, a flexible setting through the use of the telephone allows us not to give up the session and at the same time to satisfy this emotional need of the woman.
2. Equally important is the activation of a multidisciplinary care network in each phase of ART treatments. In fact, in the case presented, a forecast of any contact with a network of hospital doctors before the end of the pregnancy was lacking. After the experience of Pia and Giorgio, it is our practice in this type of pregnancies to get in touch in time with the hospital caregivers, in order to be able to communicate effectively with the healthcare workers who will follow the couples until delivery and beyond. We wanted to propose a case with a certain complexity—and not cases that foresee the working model presented here from the beginning—to highlight how important it is in this context to build an articulated project of multidisciplinary work from the beginning of taking over the couple.

## Conclusion: Business Proposal and Ethical Implications

We have tried to illustrate how the couple, in particular focusing on the woman, who receives a diagnosis of infertility/sterility and relies on an ART treatment, needs a form of multidisciplinary and integrated support and care by a team, that is, not by a single healthcare professional (the doctor) and/or another (the psychologist), but by a *group of healthcare professionals*. We retraced the various steps and related challenges that the couple faces before, during, and after the treatments, highlighting the risks of an approach that tends to separate the aspects of the body from the emotional, affective, cultural, and anthropological–social aspects of the person.

Our position has epistemological assumptions in philosophy, which today are also confirmed in the neurological sciences. In this regard, we have already mentioned the work of Pert (1997) who identified the existence of a communication network, the psychosomatic mind/body network, which runs through the body and brain. It is a non-hierarchical network that accesses all the systems of the body, which testifies to how our functioning is best expressed by an integrated mind–body entity (Brunnhuber and Michalsen, 2012), rather than by two different levels, one of which can “jump” in the other (classical psychosomatic view). Emotions pass through the body, and therefore, it becomes crucial to take them into account. On the level of ethical implications, we wish to underline that this knowledge should represent a shared heritage of all the healthcare professionals, not just a part of them, and be reflected in a clinical practice that we have tried to outline. In this perspective, it is not enough, if not harmful, to allow a desire to be realized without taking care of the results that that desire will produce. We can therefore think of the network of caregivers of ART treatments, of the multidisciplinary team, as of the system of care, external to the person, which promotes and supports for the woman and the couple that same functioning of integration (and not of division) mind–body, which Pert (1997) has identified within *organisms such as human beings*.

We have suggested this approach by generalizing a path for the couples foreseeing certain stages with the related experiences and care interventions. However, we know that each couple, precisely because they are human beings, with all the inherent vulnerabilities, lives the experience of infertility/sterility in their own and unique way. As caregivers, we need to be aware of this.

## Data Availability Statement

The original contributions presented in the study are included in the article/supplementary material, further inquiries can be directed to the corresponding author/s.

## Author Contributions

Both authors have contributed substantially to this opinion article and have approved the final version of the manuscript.

## Conflict of Interest

The authors declare that the research was conducted in the absence of any commercial or financial relationships that could be construed as a potential conflict of interest.

## Footnotes

1 Psychological work with couples undergoing ART treatments must take into account the partners’ ability to cope together with stress. Dyadic coping is a multidimensional construct, defined as the process through which partners deal with stressful situations, not as individuals, but as a couple, by leveraging on the relationship (Donato, 2014). To learn more about dyadic coping and the models that have described various aspects of it, see the review of the empirical literature and the conceptual integration published by Falconier and Rebekka (2019).

2 The World Health Organization (World Health Organization [WHO], 2020) defines sterility as the situation of a couple in which one or both members are suffering from a permanent physical condition that does not make conception possible. Infertility, on the other hand, is “a disease of the male or female reproductive system defined by the failure to achieve a pregnancy after 12 months or more of regular unprotected sexual intercourse.” The two terms, therefore, although sometimes used in common language as synonyms, refer to conditions with very different characteristics also in prognostic terms.

3 “[…] the Mind and the Body are one and the same thing which is conceived now under the attribute of Thought and now under the attribute of Extension […] the order of the actions and passions of our body is simultaneous by nature with the order of the actions and passions of the mind” (Spinoza, 1677).

4 Conrad and Schneider (1980) have used the term “medicalization” to denote the process by which certain behaviors come to be understood as questions of health and illness and therefore subject to the authority of medical institutions. Medicalization has progressed much further in highly industrialized nations than it has in less industrialized societies (Conrad, 2005, 2007). Within highly industrialized nations, the medicalization of women’s lives has proceeded at a faster pace than the medicalization of men’s lives (Inhorn, 2008).

5 The LEA are the performances and services that the National Health Service must guarantee to all citizens, free of charge or upon payment of a participation fee (the “ticket”).

6 The psychotherapist is Dr. Vasta. The first names Pia and Giorgio are fictional.

## References

[B1] Balint M. (1957). *The doctor his patient and the illness.* London: Pitman Medical Publishing.

[B2] Barrera C. M. Kawwass J. F. Boulet S. L. Nelson J. M. Perrine C. G. (2019). Fertility treatment use and breastfeeding outcomes. *Am. J. Obstet. Gynecol.* 220 .1–261e. 10.1016/j.ajog.2018.11.11007 30513338 PMC10983013

[B3] Berghuis J. P. Stanton A. L. (2002). Adjustment to a dyadic stressor: A longitudinal study of coping and depressive symptoms in infertile couples over an insemination attempt. *J. Consult. Clin. Psychol.* 70 433–438. 10.1037//0022-006x.70.2.433 11952202

[B4] Boivin J. Appleton T. C. Baetens P. Baron J. Bitzer J. Corrigan E. (2001). European Society of Human Reproduction and Embryology. Guidelines for counselling in infertility: outline version. *Hum. Rep.* 16 1301–1304. 10.1093/humrep/16.6.1301 11387309

[B5] Boivin J. Gameiro S. (2015). Evolution of psychology and counseling in infertility. *Fertil. Steril.* 104 251–259. 10.1016/j.fertnstert.2015.05.035 26092131

[B6] Boivin J. Harrison C. Mathur R. Burns G. Pericleous-Smith A. Gameiro S. (2020). Patient experiences of fertility clinic closure during the COVID-19 pandemic: appraisals, coping and emotions. *Hum. Reprod.* 35 2556–2566. 10.1093/humrep/deaa218 32761248 PMC7454659

[B7] Boivin J. Lancastle D. (2010). *Womens Health* 6 59–69. 10.2217/whe.09.79 20088730

[B8] Bottaccioli F. Bottaccioli A. G. (2017). *Psiconeuroendocrinoimmunologia e scienza della cura integrata. Il manuale. [Psychoneuroendocrinoimmunology and the science of integrated medical treatment. The manual.].* Milano: Edra.

[B9] Boz I. Özçetin E. Teskereci G. (2018). Becoming. *Guncel. Yaklasimlar Curr. Appr. Psych.* 10 506–521. 10.18863/pgy.382342

[B10] Brunnhuber S. Michalsen A. (2012). [On the relationship of psychosomatic and mind-body medicine: integrative, complementary or alternative disciplines within an evolutionary approach?] [Article in German]. *Forsch. Kompl Res. Compl. Med.* 19 86–92. 10.1159/000338537 22585105

[B11] Cavoretto P. Candiani S. Giorgione V. Inversetti A. Abu-Saba M. M. Tiberio F. (2018). Risk of spontaneous preterm birth in singleton pregnancies conceived after IVF/ICSI treatment: meta-analysis of cohort studies. *Ultras. Obstet. Gynecol.* 51 43–53. 10.1002/uog.18930 29114987

[B12] Chaves C. Canavarro M. C. Moura−Ramos M. (2019). The Role of Dyadic Coping on the Marital and Emotional Adjustment of Couples With Infertility. *Fam. Proc.* 58 509–523. 10.1111/famp.12364 29709057

[B13] Cipolletta S. Faccio E. (2013). Time experience during the assisted reproductive journey: a phenomenological analysis of Italian couples’ narratives. *J. Rep. Infant Psychol.* 31 285–298. 10.1080/02646838.2013.813627

[B14] Colucci M. (2006). Comment. Medicalizzazione. [Comment. Medicalization]. *J. Sci. Commun.* 5:2006.

[B15] Conrad P. (2005). The Shifting Engines of Medicalization. *J. Health Soc. Behav.* 46 3–14. Http://www.jstor.org/stable/4147650 (accessed January 23, 2021)., 15869117 10.1177/002214650504600102

[B16] Conrad P. (2007). *The Medicalization of Society: On the Transformation of Human Conditions into Treatable Disorders.* Baltimore: Johns Hopkins University Press.

[B17] Conrad P. Schneider J. W. (1980). *The Medicalization of Deviance: From Badness to Sickness.* St. Louis: Mosby.

[B18] Cousineau T. M. Domar A. D. (2007). Psychological impact of infertility. *Best Pract. Res. Clin. Obstet. Gynaecol.* 21 293–308. 17241818 10.1016/j.bpobgyn.2006.12.003

[B19] Cunningham J. (2017). Infertility: A primer for primary care providers. *JAAPA* 30 19–25. 10.1097/01.JAA.0000522130.01619.b7 28787288

[B20] Damasio A. (2018). *Lo strano ordine delle cose. [The strange order of things].* Milano: Adelphi.

[B21] da Motta E. L. A. Serafini P. (2002). The treatment of infertility and its historical context. *Rep Biomed.* 5 65–77. 10.1016/s1472-6483(10)61601-x 12470550

[B22] Dhillon R. Cumming C. E. Cumming D. C. (2000). Psychological well-being and coping patterns in infertile men. *Fertil. Steril.* 74 702–706. 10.1016/s0015-0282(00)01511-9 11020510

[B23] Di Trani M. (2018). ““L’intervento psicologico nell’infertilità”. [Psychological intervention in infertility],” in *In-fertilità. Un approccio multidisciplinare (p. 107-119). Atti del I Convegno nazionale. [In-fertility. A multidisciplinary approach (p. 107-119). Proceedings of the I National Conference.]*, eds Di Trani M. La Mesa A. (Rome: Sapienza Università Editrice).

[B24] Donarelli Z. Kivlighan D. M. Jr. Allegra A. Lo Coco G. (2016). How do individual attachment patterns of both members of couples affect their perceived infertility stress? An actor–partner interdependence analysis. *Pers. Individ. Differ.* 92 63–68. 10.1016/j.paid.2015.12.023

[B25] Donarelli Z. Lo Coco G. Gullo S. Marino A. Volpes A. Allegra A. (2012). Are attachment dimensions associated with infertility-related stress in couples undergoing their first IVF treatment? A study on the individual and cross-partner effect. *Hum. Reprod.* 27 3215–3225. 10.1093/humrep/des307 22926837

[B26] Donarelli Z. Salerno L. Lo Coco G. Allegra A. Marino A. Kivlighan D. M. (2019). From telescope to binoculars. Dyadic outcome resulting from psychological counselling for infertile couples undergoing ART. *J. Reprod. Inf. Psychol.* 37 13–25. 10.1080/02646838.2018.1548757 30468393

[B27] Donato S. (2014). Il coping diadico, ovvero far fronte allo stress insieme: una rassegna della letteratura [*Dyadic coping*, that is *managing stress together: A review of the literature*]. *G. Ital. di Psicol.* 3 473–504. 10.1421/78499

[B28] Doss B. D. Rhoades G. K. (2017). The transition to parenthood: impact on couples’ romantic relationships. *Curr. Opin. Psychol.* 13 25–28. 10.1016/j.copsyc.2016.04.003 28813289

[B29] Engel G. L. (1977). The Need for a New Medical Model: A Challenge for Biomedicine. *Science* 196 129–136. 847460 10.1126/science.847460

[B30] Falconier M. K. Rebekka K. (2019). Dyadic Coping in Couples: A Conceptual Integration and a Review of the Empirical Literature. *Front. Psychol.* 10:571. 10.3389/fpsyg.2019.00571 30971968 PMC6443825

[B31] Foucault M. (1963). *The Birth of the Clinic: An Archaeology of Medical Perception.* Oxfordshire, UK: Taylor & Francis e-Library.

[B32] Frederiksen Y. Farver-Vestergaard I. Skovgård N. G. Ingerslev H. J. Zachariae R. (2015). Efficacy of psychosocial interventions for psychological and pregnancy outcomes in infertile women and men: a systematic review and meta-analysis. *BMJ Open* 5:e006592. 10.1136/bmjopen-2014-006592 25631310 PMC4316425

[B33] Gameiro S. Boivin J. Dancet E. de Klerk C. Emery M. Lewis-Jones C. (2015). ESHRE guideline for routine psychosocial care in infertility and medically assisted reproduction. *Hum. Reprod.* 30 2476–2485. 10.1093/humrep/dev177 26345684

[B34] Gardner W. L. Rotella K. N. Nikolovski J. (2020). Implicit Maternal Intuition Confidence Is Associated With Maternal Well-Being Across Cultures. *Front. Psychol.* 11:289. 10.3389/fpsyg.2020.00289 32153480 PMC7047325

[B35] Greil A. L. McQuillan J. Slauson-Blevins K. S. (2011). *The Social Construction of Infertility. Sociology Department, Faculty Publications.* 655. Available online at: Https://digitalcommons.unl.edu/sociologyfacpub/655 (accessed on January 23, 2021).

[B36] Ha J. Y. Ban S. H. (2020). Effect of resilience on infertile couples’ quality of life: an actor-partner interdependence model approach. *Health Qual. Life Outcomes* 18:295. 10.1186/s12955-020-01550-6 32873294 PMC7466783

[B37] Hocaoglu C. (2018). ““The Psychosocial Aspect of Infertility”,” in *Infertility, Assisted Reproductive Technologies and Hormone Assays. London: IntechOpen Limited, July 17th 2019*, ed. Dhastagir S. S.. Available online at: 10.5772/intechopen.80713 (accessed on January 23, 2021).

[B38] Inhorn M. C. (2008). Defining women’s health: a dozen messages from more than 150 ethnographies. *Med. Anthropol. Q.* 20 345–378. 10.1525/maq.2006.20.3.345 16937621

[B39] Iyengar U. Rajhans P. Fonagy P. Strathearn L. Kim S. (2019). Unresolved Trauma and Reorganization in Mothers: Attachment and Neuroscience Perspectives. *Front. Psychol.* 10:110. 10.3389/fpsyg.2019.00110 30761051 PMC6363675

[B40] Khetarpal A. Singh S. (2012). Infertility: Why can’t we classify this inability as disability? *Austr. Med. J.* 5 334–339. 10.4066/AMJ.2012.1290 22848333 PMC3395292

[B41] Laney E. K. Carruthers L. Hall M. E. L. Anderson T. L. (2014). Expanding the Self: Motherhood and Identity Development in Faculty Women. *J. Fam. Issues* 35 1227–1251. 10.1177/0192513X13479573

[B42] Laney E. K. Hall M. E. L. Anderson T. L. Willingham M. M. (2015). Becoming a Mother: The Influence of Motherhood on Women’s Identity Development. *Identity* 15 126–145. 10.1080/15283488.2015.1023440

[B43] Li Y. Zhang X. Shi M. Guo S. Wang L. (2019). Resilience acts as a moderator in the relationship between infertility-related stress and fertility quality of life among women with infertility: a cross-sectional study. *Health Qual. Life Outcomes* 17:38. 10.1186/s12955-019-1099-8 30770738 PMC6377764

[B44] Lindsay T. J. Vitrikas K. R. (2015). Evaluation and Treatment of Infertility. *Am. Fam. Physician* 91 308–314. Https://www.aafp.org/ (accessed January 23, 2021), 25822387

[B45] Luk B. H. Loke A. Y. (2015). The Impact of Infertility on the Psychological Well-Being, Marital Relationships, Sexual Relationships, and Quality of Life of Couples: A Systematic Review. *J. Sex Marital Ther*. 41 610–625. 10.1080/0092623X.2014.958789 25211377

[B46] Luk B. H. Loke A. Y. (2019). Sexual satisfaction, intimacy and relationship of couples undergoing infertility treatment. *J. Reprod. Infant Psychol.* 37 108–122. 10.1080/02646838.2018.1529407 30317866

[B47] Martins M. V. Costa P. Peterson B. D. Costa M. E. Schmidt L. (2014a). Marital stability and repartnering: infertility-related stress trajectories of unsuccessful fertility treatment. *Fertil. Steril.* 102 1716–1722. 10.1016/j.fertnstert.2014.09.007 25439808

[B48] Martins M. V. Peterson B. D. Almeida V. Mesquita-Guimarães J. Costa M. E. (2014b). Dyadic dynamics of perceived social support in couples facing infertility. *Hum. Reprod.* 29 83–89. 10.1093/humrep/det403 24218401

[B49] Märtsin M. (2018). Becoming an employed mother: Conceptualising adult identity development through semiotic cultural lens. Women’s Stud. *Int. Forum* 68 11–18.

[B50] Mohiyiddeen L. Cerra C. (2017). “Biopsychosocial Aspects of Infertility,” in *Biopsychosocial Factors in Obstetrics and Gynaecology*, eds Edozien L. O’Brien P. (Cambridge: Cambridge University Press), 110–120.

[B51] Molgora S. Baldini M. P. Tamanza G. Somigliana E. Saita E. (2020). Individual and Relational Well-Being at the Start of an ART Treatment: A Focus on Partners’. *Gender Diff. Front. Psychol.* 11:2027. 10.3389/fpsyg.2020.02027 PMC7549400 33117204

[B52] Molgora S. Fenaroli V. Acquati C. De Donno A. Baldini M. P. Saita E. (2019). Examining the Role of Dyadic Coping on the Marital Adjustment of Couples Undergoing Assisted Reproductive Technology (ART). *Front. Psychol.* 10:415. 10.3389/fpsyg.2019.00415 30906270 PMC6418016

[B53] Moura-Ramos M. Santos T. A. Canavarro M. C. (2017). The Role of Attachment Anxiety and Attachment Avoidance on the Psychosocial Well-being of Infertile Couples. *J. Clin. Psychol. Med. Settings* 24 132–143. 10.1007/s10880-017-9496-9 28536903

[B54] Namdar A. Naghizadeh M. M. Zamani M. Yaghmaei F. Sameni M. H. (2017). Quality of life and general health of infertile women. *Health Qual. Life Outcomes* 15:139. 10.1186/s12955-017-0712-y 28701163 PMC5508693

[B55] Ngai F. W. Loke A. Y. (2021). Relationships between infertility-related stress, family sense of coherence and quality of life of couples with infertility. *Hum. Fertil.* 12 1–13. 10.1080/14647273.2021.1871781 33432870

[B56] Ockhuijsen H. D. van den Hoogen A. Macklon N. S. Boivin J. (2013). The PRCI study: design of a randomized clinical trial to evaluate a coping intervention for medical waiting periods used by women undergoing a fertility treatment. *BMC Women’s Health* 13:35. 10.1186/1472-6874-13-35 PMC3766696 24004640

[B57] Ongaro Basaglia F. (2012). *Salute/Malattia. Le parole della medicina. [Health / Illness. The words of medicine].* Merano and Bolzano: Alpha & Beta.

[B58] Pacilli M. G. (2019). *Uomini duri. Il lato oscuro della mascolinità. [Tough men. The dark side of masculinity].* Bologna: Il Mulino.

[B59] Patel A. Sharma P. S. V. N. Kumar P. (2018). In cycles of dreams, despair, and desperation:” Research perspectives on infertility specific distress in patients undergoing fertility treatments. *J. Hum. Reprod. Sci.* 11 320–328. 10.4103/jhrs.JHRS_42_18 30787515 PMC6333040

[B60] Pauli W. (1952). *Psiche e natura. [The Interpretation of Nature and the Psyche].* Milan: Adelphi, 2006.

[B61] Pert C. B. (1997). *Molecules of Emotion: Why You Feel the Way You Feel.* New York, NY: Scribner.

[B62] Procaccini C. Pucino V. De Rosa V. Marone G. Matarrese G. (2014). Neuroendocrine networks controlling immune system in health and disease. *Front. Immunol.* 5:143. 10.3389/fimmu.2014.00143 24778633 PMC3985001

[B63] Rosner M. (2012). *Recovery From Traumatic Loss: A Study Of Women Living Without Children After Infertility. Doctorate in Social Work (DSW) Dissertations. 20. University of Pennsylvania: ScholarlyCommons.* Available online at: https://repository.upenn.edu/edissertations_sp2/20 (accessed on November 23, 2020).

[B64] Salerno A. Piccolo C. (2006). “Il corpo smarrito: ridefinizione dell’identitaÌ corporea nella coppia sterile,” in *Il fascino discreto della famiglia. Mutazioni familiari e nuove competenze*, eds Di Vita A. M. Garo M. (Milan: Franco Angeli), 122–150.

[B65] Schirone T. (2013). Identità e trasformazione di identità: la maternità [Identity and identity transformation: motherhood]. *Studi Urbinati, B-Scienze umane e sociali* 80 189–195.

[B66] Solano L. (2016). Al di laÌ di Cartesio. *Riv. Psicoanal.* 62 49–72.

[B67] Solano L. (2018). “Il rapporto corpo-mente e la qualità delle relazioni nella costruzione della salute”. [The body-mind relationship and the quality of relationships in the construction of health],” in *In-fertilità. Un approccio multidisciplinare (p. 9-21). Atti del I Convegno nazionale. [In-fertility. A multidisciplinary approach (p. 9-21). Proceedings of the I National Conference]. Rome, May 5-6 2017*, eds Di Trani M. La Mesa A. (Rome: Sapienza Università Editrice).

[B68] Sparzani A. Panepucci A. (eds) (2016). *Jung e Pauli. Il carteggio originale: l’incontro tra Psiche e Materia. [Jung and Pauli. The original correspondence: the encounter between Psyche and Matter].* Bergamo: Moretti & Vitali.

[B69] Spinoza B. (1677). *Etica dimostrata secondo l’ordine geometrico. Parte terza: della natura e dell’origine degli affetti. [Ethics demonstrated in geometrical order. Third part: the nature and origin of the emotions].* Rome: Editori Riuniti University Press, 2019.

[B70] Stern D. N. (1995). *The Motherhood Constellation: A Unified View of Parent-Infant Psychotherapy*. London and. New York: Routledge, 2018.

[B71] Stern D. N. Bruschweiler-Stern N. Freeland A. (1998). *The Birth of a Mother: How the Motherhood Experience Changes You Forever.* New York: Basic Books.

[B72] Thorn P. (2009). Understanding Infertility: Psychological and Social Considerations from a Counselling Perspective. *Int. J. Fertil. Steril* 3 48–51.

[B73] Van den Broeck U. D’Hooghe T. Enzlin P. Demyttenaere K. (2010). Predictors of psychological distress in patients starting IVF treatment: infertility-specific versus general psychological characteristics. *Hum. Reprod.* 25 1471–1480. 20338959 10.1093/humrep/deq030

[B74] Vasta F. N. (2020a). *“Quale etica per lo psicoterapeuta che lavora con le coppie.* Available online at: https://siru-course.eminerva.eu/home.php#program (accessed on January 23, 2021).

[B75] Vasta F. N. (2020b). *We are all Citizens of Coronaville: Psychological Reflections on Coronavirus in Italy. Interview given to D. Polito. March 20, 2020. Published on the website of the American Group Psychotherapy Association (AGPA).* Available online at: https://www.agpa.org/docs/default-source/practice-resources/we-are-all-citizens-of-coronaville.pdf?sfvrsn=d1f09ba9_0 (accessed on January 23, 2021)

[B76] Vasta F. N. Girelli R. (2016). “Infertilità, procreazione medicalmente assistita, prematurità. [Infertility, medically assisted reproduction, preterm birth],” in *Ferite [Wounds]. La camera blu. Rivista di studi di genere*, Vol. 14 ed. Margherita G. 216–245.

[B77] Vasta F. N. Girelli R. (2017). *PMA e prematurità: l’intervento psicologico con le coppie di genitori in terapia intensiva neonatale. Relazione presentata al I Congresso Nazionale della Società Italiana della Riproduzione Umana. [ART and prematurity: psychological intervention with couples of parents in neonatal intensive care. Report presented at the I National Congress of the Italian Society of Human Reproduction].* Rome: Alpes.

[B78] Vasta F. N. Girelli R. (2019). *La rappresentazione del corpo femminile nei percorsi di PMA: una lettura psicodinamica. Relazione presentata al III Congresso Nazionale della Società Italiana della Riproduzione Umana. [The representation of the female body in the paths of ART: a psychodynamic reading. Report presented at the III National Congress of the Italian Society of Human Reproduction].* Rome: Alpes..

[B79] Vasta F. N. Girelli R. Aprea A. (2013). “Il lavoro psicologico in terapia intensiva neonatale: proposta di un modello di intervento gruppale. [Psychological work in neonatal intensive care: proposal of a group intervention model],” in *Quale omogeneità nei gruppi? Elementi di teoria, clinica e ricerca*, eds Vasta F. N. Girelli R. Gullo S. (Rome: Alpes), 237–254.

[B80] Vernero S. (2017). *“Sovrautilizzo: esami e trattamenti”. [Overuse: tests and treatments]. In: AAVV: Slow Medicine. Le parole della medicina che cambia (p. 110-113). [Slow Medicine. The words of medicine that changes].* Rome: Pensiero Scientifico Editore.

[B81] Walentynowicz-Moryl K. (2020). In Front of the Mirror of Social Expectations: Experiences of Women Until They Are Given a Diagnosis of Infertility. *Przegla?d Socjol. Jakosìciowej* 16 66–83. 10.18778/1733-8069.16.1.05

[B82] World Health Organization [WHO] (2020). *Infertility.* Available online at: Https://www.who.int (accessed on January 23, 2021).

[B83] Zurlo M. C. Cattaneo Della, Volta M. F. Vallone F. (2018). Predictors of quality of life and psychological health in infertile couples: the moderating role of duration of infertility. *Qual. Life Res.* 27 945–954. 10.1007/s11136-017-1781-4 29307056

[B84] Zurlo M. C. Cattaneo Della, Volta M. F. Vallone F. (2019). The association between stressful life events and perceived quality of life among women attending infertility treatments: the moderating role of coping strategies and perceived couple’s dyadic adjustment. *BMC Public Health* 19:1548. 10.1186/s12889-019-7925-4 PMC6873711 31752817

[B85] Zurlo M. C. Cattaneo Della, Volta M. F. Vallone F. (2020). Infertility-Related Stress and Psychological Health Outcomes in Infertile Couples Undergoing Medical Treatments: Testing a Multi-dimensional Model. *J. Clin. Psychol. Med. Sett.* 27 662–676. 10.1007/s10880-019-09653-z 31471847

